# How to look after and care for a slit lamp

**Published:** 2010-09

**Authors:** Ismael Cordero

**Affiliations:** Senior Clinical Engineer, ORBIS International, 520 8th Avenue, 11th Floor, New York, NY 10018, USA.

**Figure:**
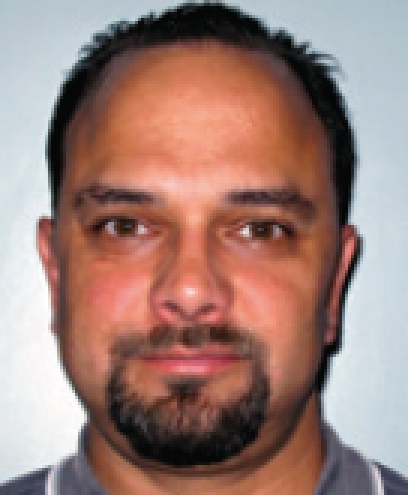


The slit lamp is an essential and often-used diagnostic instrument in ophthalmology. It provides illumination and magnification for the examination of many structures of the anterior segment. With complementary lenses, it is also used to examine the chamber angle and a significant part of the retina. Its name derives from the fact that a narrow slit of light is used to illuminate the various structures being examined.

By following these simple suggestions, you can ensure that a slit lamp performs optimally and remains functional for longer.

## Location

- Place the slit lamp where it is easily accessible to both staff and patients, some of whom may have physical disabilities.
- An electrical outlet should be available nearby and the power cord should not be in the path of staff or patients.
- The slit lamp should not be exposed to excessive temperature extremes, such as those produced by direct sunlight or air conditioning.
- The slit lamp should be kept in a dry environment since there could be fungal growth (mould or mildew) on the optical components if they are exposed to humidity (combined heat and moisture).

## Spare parts

- Spare bulbs and fuses should be kept within easy reach in order to avoid delays in patient care.
- The minimum recommended stock of bulbs and fuses is two of each per slit lamp.
- When a part is used, it should be restocked immediately.

## Replacing the bulb

- When handling or replacing a bulb, take care not to leave fingerprints on the bulb. Oil from your fingers can create hot spots on the bulb which will reduce its life. As a rule, handle bulbs with paper tissue or with cotton gloves.
- Check that you replace the bulb housing in the right position; otherwise the quality of the slit beam is compromised. Adjusting the position of the housing may correct a distorted beam.

## Cleaning

- The slit lamp should be cleaned weekly, at a minimum, or more often if in a dusty environment.
- The slit lamp housing should be cleaned with a cloth that has been slightly dampened with water. No other liquids or corrosive agents should be used.
- The exposed surfaces of the eyepiece optics (1) and the objective lens (2) should be cleaned using a soft optical dust brush. If, after being dusted, they still need additional cleaning, the lenses should then be wiped carefully with a lens cleaning cloth or with cotton swabs and lens cleaning solution.

## Operational checks

The following functions should be checked **weekly.** The hospital's maintenance team or the service agent should be called if any problems are noticed during these checks.

- Brightness control: should noticeably vary the bulb's brightness.
- Table top movement: should move up and down freely.
- Chin rest adjustment (3): should move up and down freely.
- Joystick (4): should provide smooth motion up and down, forward and backward, and left and right.
- Slit controls: should smoothly vary the slit width (5), length (6), and inclination (6).
- Illumination rotation arm (7): should move smoothly and lock into position with the locking screw (8).
- Microscope rotation arm (9): should move smoothly and lock into position with the locking screw (10).
- Illumination tilting latch (11): should vary the illumination angle in stages.
- Filter changing knob (12): should change the filters.
- Magnification lever (13): should switch the magnification.
- The mechanism just behind the objectives that adjusts the pupillary distance (14) should move smoothly.

## Other tips for care and maintenance

- If the clinic is subject to voltage fluctuations, the slit lamp should be plugged into a voltage stabiliser.
- When examining several patients in a row, the illumination should be maintained at a low level rather than switching it off between patients and then on again for each patient. This prolongs the bulb's life.
- Moving the slit lamp should be avoided when the bulb is hot because the hot filament is more likely to break.
- When not in use, the slit lamp should be covered with its plastic dust cover. If not provided, a simple cover can be made out of cloth - the thicker/denser the better.
- If the slit lamp is stored in an environment prone to humidity, keep a sachet of silica gel drying agent or fungicidal (anti-mould) pellets within the dust cover, or use a dehumidifier in the room.
- The forward and backward, and left and right movements of the slit lamp rely on the joystick (4), a rod (15) connecting the two geared wheels (16), and the two rails (17) which support the wheels. These mechanical devices may seize up and affect the smooth operation of the slit lamp. If this is the case, apply a light oil spray, such as WD40, to a piece of paper tissue and use the tissue to wipe the rod, the pad under the joystick (18), the wheels, and the rails. This should solve the problem. Oil should never be sprayed directly onto these parts.

**New Series**

This is the first installment in a series on practical equipment care, maintenance, and repair.

**Figure 1:**
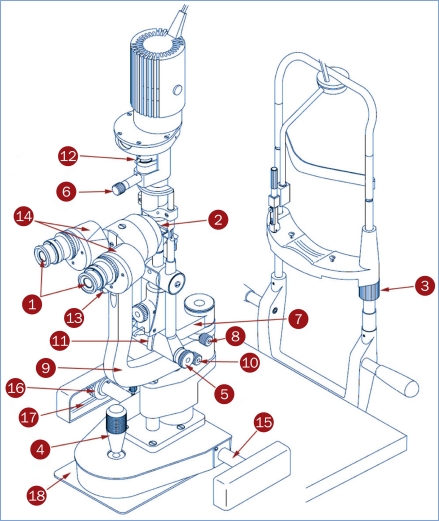
Diagram of a Haag-Streit style slit lamp

